# From mucus secretion to immune surveillance: the evolving roles of goblet cells in intestinal homeostasis and inflammation

**DOI:** 10.3389/fimmu.2026.1735230

**Published:** 2026-04-07

**Authors:** Ning Du, Kun Zhang, Jing Guo, Wenlong Yan, Lingfen Xu

**Affiliations:** Department of Pediatrics, Shengjing Hospital of China Medical University, Shenyang, Liaoning, China

**Keywords:** intestinal goblet cells, development, function, intestinal barriers, inflammatory bowel disease

## Abstract

**Background:**

Intestinal goblet cells (GCs) are specialized epithelial cells essential for forming the protective mucus barrier. Recent research has significantly expanded our understanding of their roles beyond mucus secretion, revealing critical functions in immune regulation and mucosal homeostasis. Dysfunction of these cells is implicated in the pathogenesis of intestinal disorders, particularly inflammatory bowel disease (IBD).

**Scope and methods:**

This review synthesizes the current literature on intestinal GC biology, encompassing their developmental pathways, cellular heterogeneity, and multifaceted functions within the intestinal chemical, mechanical, immune, and biological barriers.

**Key findings and contribution:**

We highlight the remarkable plasticity and heterogeneity of GCs, detailing newly identified subtypes such as sentinel GCs and intercrypt GCs, and their distinct roles in mucosal defence. The review elucidates how GCs contribute to intestinal homeostasis not only through mucin (*MUC2*) production but also via antigen sampling, cytokine secretion, and interactions with the commensal microbiota. Furthermore, we consolidate evidence on the signalling pathways and molecular regulators governing GC differentiation and function.

**Conclusion and implications:**

The dysfunction or depletion of intestinal GCs is a hallmark of IBD, leading to barrier breakdown and sustained inflammation. This review underscores the emerging role of GCs as key guardians of intestinal health and promising therapeutic targets. A deeper understanding of GC biology paves the way for novel strategies aimed at restoring intestinal barrier function in IBD.

## 1 Introduction

The intestine is a complex and highly specialized epithelial barrier that plays a role in immune regulation, nutrient absorption, and barrier protection, all of which is crucial for maintaining intestinal homeostasis (1, 2). Intestinal epithelial cells, as a primary component of the intestinal barrier, perceive stimuli from the external environment and respond to them accordingly (3). As a type of specialized epithelial cells, intestinal goblet cells secrete mucin and water to form a protective mucus layer, serving as key components of the intestinal mechanical barrier; recent studies highlight their essential role in both intestinal and immune barrier function (4–7). Abnormalities in the number and function of intestinal goblet cells cause intestinal barrier dysfunction and predispose to the development of various intestinal diseases, and over the past few years, intestinal goblet cells have emerged as a core player in the regulation of intestinal barrier function (8–10).

In this review, we discuss the development of intestinal goblet cells, various influencing factors, and their roles in the development of inflammatory bowel disease (IBD).

## 2 Development of intestinal goblet cells

**Figure 1:**
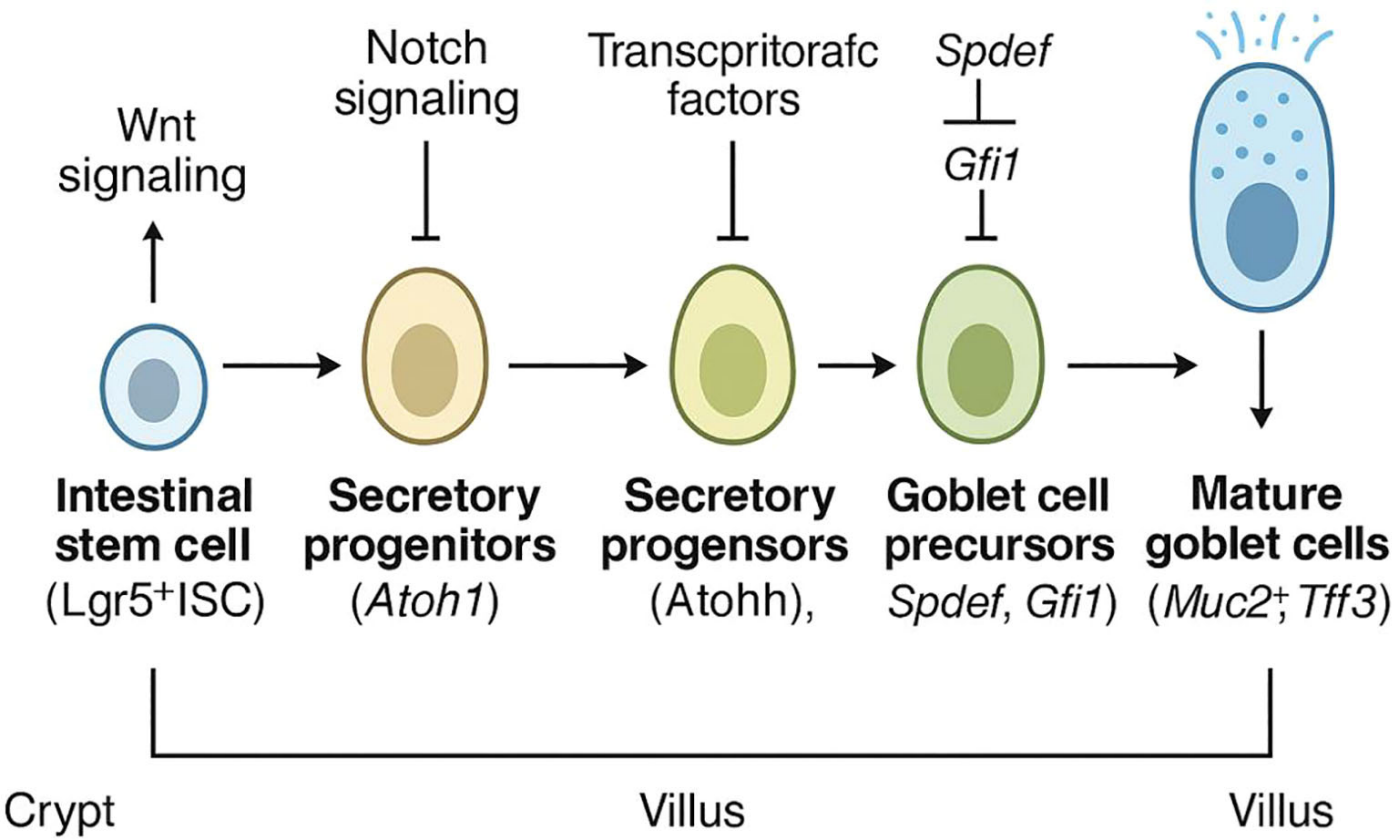
Development of intestinal goblet cells. Through signalling pathways orchestrated by Wnt and Notch, along with specific transcription factors, these stem cells commit to the secretory lineage and mature into functional goblet cells located in the villi, which are capable of mucin secretion.

As a semi-permeable and highly regenerative tissue, the intestine primarily functions in digesting and absorbing ingested nutrients (11, 12). While performing its functions, the gut is constantly exposed to the external environment, necessitating the frequent renewal of the epithelial tissue to protect the gut against the invasion of various harmful substances and the occurrence of different intestinal diseases, a process accomplished by the proliferation of adult stem cells in the basal part of the crypts of the intestinal mucosa (13, 14). These adult stem cells differentiate into cell types with different functions during migration from the base of the crypt to the top and comprise two primary groups: the absorptive lineage, which includes enterocytes and microfold cells, and the secretory lineage, which includes goblet, Paneth, enteroendocrine, and Tuft cells (15–24). These intestinal cells collaborate to maintain intestinal homeostasis. Among them, the goblet cells are named for their resemblance to a cup, and there is a gradient in their distribution along the intestine, gradually increasing from near to far, with the duodenum having the lowest and the rectum exhibiting the highest (25, 26). Of the intestinal secretory lineage cells, goblet cells are the most abundantly produced (27). Intestinal goblet cells are secretory cells that originate from the bottom of the intestinal crypt (28). Within the crypt, crypt base columnar cells split successively to produce progenitor cells, which divide and proliferate rapidly after entering the transit expansion zone before differentiating and developing into mature goblet cells (28, 29). The differentiation of intestinal goblet cells is primarily regulated by signalling pathways such as those of Notch, Wnt/β-catenin, Hippo, and epidermal growth factor (EGF), whereas their early differentiation is primarily regulated by molecules such as *atonal bHLH transcription factor 1* (*Atoh1*), and molecules such as *Krüppel-like factor 4* (*KLF4*) and *the SAM pointed domain ETS factor* (*SPDEF*) primarily mediate their terminal differentiation (11, 30–35). Intestinal goblet cells are a class of short-lived cells that are renewed once every 7 days and play a crucial protective role in the intestinal barrier by producing and secreting mucus and releasing antimicrobial substances (26) (**Figure 1**).

## 3 Classification of intestinal goblet cells

Intestinal goblet cells were long thought to be a homogeneous population of secretory cells; however, advances in science and technology have revealed a greater degree of heterogeneity than previously appreciated (36) (**Table 1**). Birchenough et al. (37) analysed mouse colon tissues and identified a subtype of intestinal goblet cells in the upper crypt named sentinel goblet cells (senGCs). Located at the opening of colonic crypts, sentinel goblet cells (senGCs) are a specialized subset that can uniquely endocytose ligands of Toll-like receptors (TLRs), including agonists for TLR1/2, TLR4, and TLR5. The internalization of ligands activates these cells through the nod-like receptor family pyrin domain containing 6 (NLRP6), causing a swift, calcium-dependent secretion of stored mucin 2 (MUC2) from senGCs. Simultaneously, activated senGCs emit intercellular signals that cause adjacent responding goblet cells to release MUC2. The synchronized release of mucus from senGCs and nearby goblet cells helps to physically remove bacteria from the colonic crypts. This defence mechanism strengthens the intestinal mucus barrier, preventing bacterial invasion and reducing the risk of mucosal inflammation, which helps maintain intestinal balance (37).

**Table 1:** Classification of intestinal goblet cells and function.

Classification	Name	Description	Function	Ref.
Classification according to development	Canonical goblet cells	Predominantly express the known goblet cells specific genes *Atoh1*, *Clca1*^c^, *MUC2*^d^, and *Fcgbp*^e^	Related to secretion, glycosylation, and endoplasmic reticulum stress	(36, 38–40)
	Noncanonical goblet cells	Mainly express the genes Hes1^f^, *Gsdmc4*^g^, *Dmbt1*^h^, *MUC17*^i^, and ion channels related to intestinal cells	Related to lipid and amino acid metabolism, detoxification, and intestinal absorption	(36, 40)
Classification according to the expression of markers	Typical goblet cells	Expressing the typical cuprocyte-associated genes Clca1^c^ and Fcgbp^e^	-	(42)
	Proliferative goblet cells	Expressing the proliferative marker *Mki67*	-	(36)
	Nontypical goblet cells	Expressing the nontypical goblet cells-related genes *Gsdmc4*^g^, *Dmbt1*^h^, and *Sis*	-	(36)
	Fully differentiated goblet cells	Expressing the fully differentiated cell-associated gene *Mxd1*^j^	-	(36, 43)
Classification according to the location	icGCs^a^	Surface epithelium located between crypts; high mucus turnover	Involved in stress, cell differentiation, apoptosis, and response to protein translocation	(36)
	senGCs^b^	Locate at the opening of the crypt	Rapidly produce and secrete a more permeable intercryptal mucus	(37)
	Crypt-residing goblet cells	Reside in the crypt	Release stored mucus after receiving a signal to protect the crypts from damage	(36)

^a^ intercrypt goblet cells; ^b^ sentinel goblet cells; ^c^ calcium-activated chloride channel regulator 1; ^d^ mucin 2; ^e^ IgGFc-binding protein; ^f^ hairy and enhancer of split-1; ^g^ Gasdermin 4; ^h^ deleted in malignant brain tumors 1; ^i^ mucin 17; ^j^ MAX dimerisation protein 1.

Nyström et al. (36) revealed a dynamic system comprising different goblet cell subtypes by analysing mouse small intestinal and colonic cells expressing *MUC2* and identified a unique differentiation trajectory and maturation process of goblet cells, which were divided into two distinct and mutually independent trajectories that included canonical goblet cells (canonical GCs) and noncanonical goblet cells (noncanonical GCs), with both trajectories originating from proliferating cells in the lower crypt and forming two small populations of goblet cells in the surface epithelium with high *Schlafen 4* or *aquaporin-8* expression. The canonical GCs predominantly express goblet cell-specific genes *Atoh1*, *calcium-activated chloride channel regulator 1* (*Clca1*), *MUC2*, and *IgGFc-binding protein* (*Fcgbp*), whereas noncanonical GCs primarily express the genes *Hes1*, *Gasdermin 4* (*Gsdmc4*), *deleted in malignant brain tumors 1* (*Dmbt1*), *mucin 17*, and ion channels related to intestinal cells, which are reportedly specific to goblet and intestinal cells, respectively, in previous related studies (38–40). Similarly, this study demonstrated that canonical GCs have functions related to secretion, glycosylation, and endoplasmic reticulum stress, whereas noncanonical GCs function in lipid and amino acid metabolisms, detoxification, and intestinal absorption, improving the understanding of the functions of the different goblet cell subtypes (36) (**Table 1**). In analysing the single-cell RNA sequencing of mouse intestinal goblet cells, small intestinal and colon goblet cells were classified into nine and eight clusters, respectively, and these intestinal goblet cells were classified into four primary types according to their expressed markers: typical goblet cells expressing the typical cuprocyte-associated genes *Clca1* and *Fcgbp*, proliferative goblet cells expressing the proliferative marker *Mki67*, nontypical goblet cells expressing the atypical goblet cell-related genes *Gsdmc4*, *Dmbt1*, and *Sis*, and fully differentiated goblet cells expressing the fully differentiated cell-associated gene *MAX dimerisation protein 1 (*36, 41–43)(**Table 1**). These intestinal goblet cells may have been formed during the differentiation process, and the relationships between the different goblet cell clusters indicate the potential plasticity of intestinal goblet cells.

**Figure 2:**
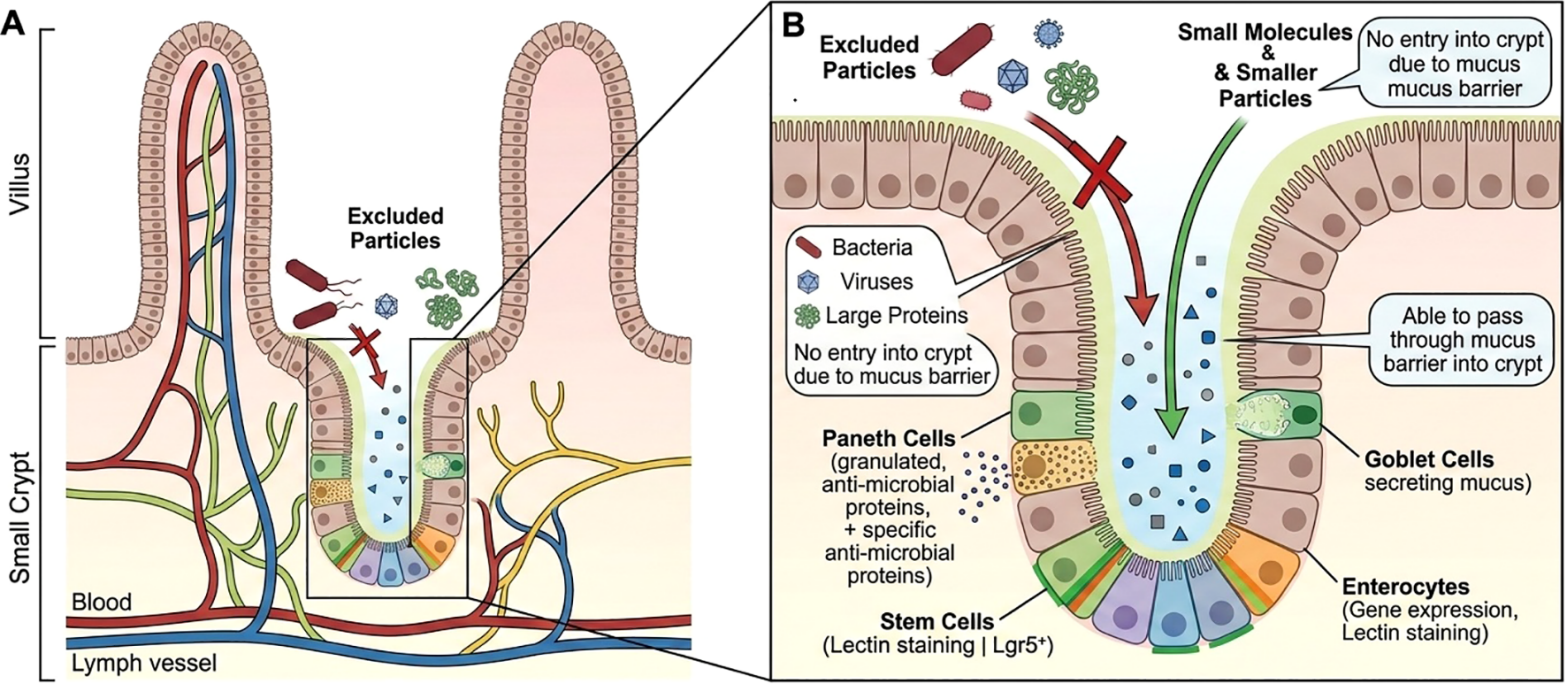
Differential exclusion in the intestinal crypt. Illustration of the intestinal crypt-villus axis depicting the mucus layer’s role as a size-selective barrier. **(A)** An overview of the villus-crypt architecture depicting the anatomical relationship among the villus epithelium, crypt, and the underlying blood and lymphatic vessels. **(B)** Detailed illustration of the crypt compartment highlighting cellular composition and the mechanism of mucus-mediated exclusion.

In addition, goblet cells of a previously unrecognized and functionally distinct subtype were identified and named intercrypt goblet cells (icGCs) (36). icGCs include all goblet cells on the crypt surface, primarily comprising the most differentiated canonical GCs, and these intestinal goblet cells have a distinctive morphology and specific gene expression profiles that can be localized with the *wheat germ agglutinin lectin* and be detected using *non-O glycosylated MUC2* antiserum (36). *Wheat germ agglutinin* and *Ulex europaeus agglutinin 1* immunofluorescence double staining revealed that the mucus secreted by icGCs is primarily in the spatial region around the crypt plumes at the top of the colonic crypt openings and is called the intercrypt mucus (36). The intercrypt mucus keeps out bacteria of size 1 μm but not smaller molecules, while the crypt plume mucus is impenetrable to 1 μm-sized bacteria and smaller particles, and the two form a dense mesh structure on the mucosal surface, blocking the invasion of bacteria and other harmful substances and enhancing intestinal mucus homeostasis (36) (**Figure 2**). The mechanism of how intestinal goblet cells utilize the same set of core proteins to produce mucus of such different properties is poorly understood; however, the differences may be related to mucus processing, and further research is urgent (25) (**Table 1**). The identification of senGCs/icGCs creates new avenues for developing targeted therapies aimed at fortifying the crypt barrier against bacterial invasion in IBD.

## 4 Functions of intestinal goblet cells

As a special organ in direct contact with the external environment, the intestinal tract has been constantly subjected to various stimulations; thus, the formation of the intestinal barrier is essential to maintain intestinal homeostasis (44). The primary intestinal barriers include chemical, mechanical, immune, and biological forms (45). The intestinal goblet cells, as members of the intestinal epithelium, are crucial in the function and maintenance of the intestinal barrier.

### 4.1 Role of intestinal goblet cells in the chemical barrier

**Figure 3:**
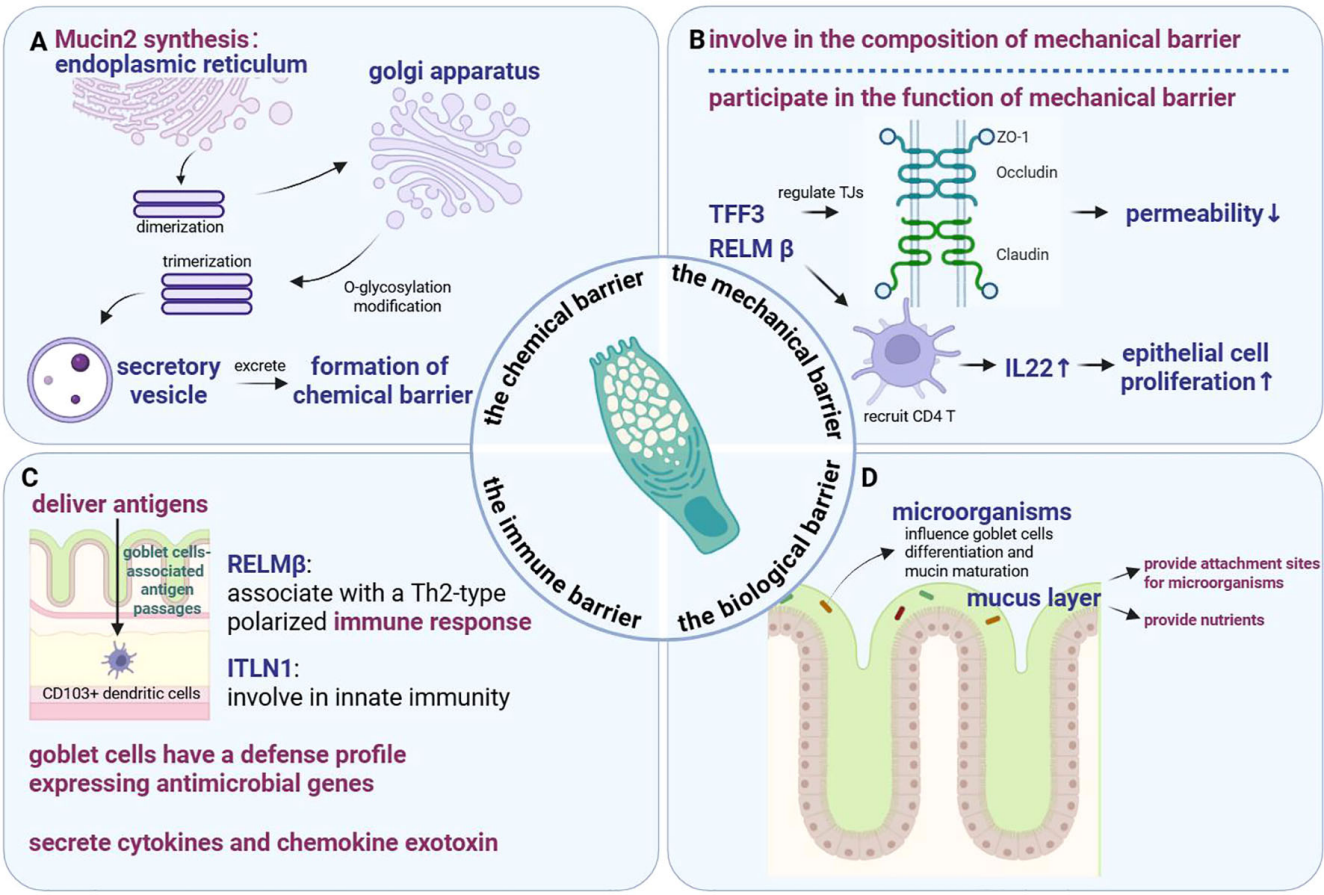
Functions of intestinal goblet cells. **(A)** The role of the intestinal goblet cells in the chemical barrier. **(B)**. The role of the intestinal goblet cells in the mechanical barrier. **(C)** The role of the intestinal goblet cells in the immune barrier. **(D)** The role of the intestinal goblet cells in the biological barrier. TFF3, Trefoil factor 3; RELM β, Resistin-like molecule β; TJs, Tight junctions; ZO-1, Zonula occludens 1; IL22, Interleukin 22; ITLN1, Interectin-1.

The intestinal chemical barrier, also known as the mucus barrier, primarily comprises mucus secreted by the epithelial cells of the intestinal mucosa, mucin, and various digestive enzymes, lysozyme, as well as bacteriostatic substances secreted by beneficial intestinal bacteria, which constitute the first defence line for the intestine against invasion by harmful luminal contents (46–48). Mucus is primarily synthesized and secreted by goblet cells and covers the surface of epithelial cells (46, 49–51). *MUC2* is the primary component of mucus that serves as a skeleton, which is first dimerized in the intestinal goblet cell endoplasmic reticulum to form a dimer through intermolecular disulfide bonding and subsequently transferred to the Golgi to complete O-glycosylation modification; the *MUC2* dimer is transformed into a trimer in the trans-Golgi network and finally encapsulated within secretory vesicles (52–54). In addition to *MUC2*, this secretory vesicle contains an *Fc fragment of IgG-binding protein*, *chloride channel accessory*, *zymogen granule protein*, and *anterior gradient homologue 2 (*52). During the upward movement of the goblet cells from the crypt base, this vesicle is continuously filled and consequently fuses with the apical membrane of the goblet cells, facilitating the excretion of its contents through the cytotoxic action of the goblet cells (52). Finally, for the accumulated mucin to be sufficiently swollen to form the dense meshwork, the mucin requires a high pH, low Ca^2+^ environment, and HCO_3_^• -^ supplied by the cystic fibrosis transmembrane conductance regulator channel, which forms a dense meshwork that segregates the bacteria from the intestine by frequently renewing itself, preventing the bacterial invasion of the intestine (3, 46, 52, 55–59). Han et al. (60) found that β-hydroxybenzoic acid enhanced the function of the intestinal chemical barrier by increasing the levels of intestinal goblet cells and *MUC2*, which effectively alleviated DSS-induced colonic inflammation (**Figure 3a**). Therefore, intestinal goblet cells are vital in the maintenance and function of the intestinal chemical barrier.

### 4.2 Role of intestinal goblet cells in the mechanical barrier

The intestinal mechanical barrier, also known as the physical barrier, primarily comprises intestinal epithelial cells and the tightly connected proteins between them, which protect the intestine against the invasion of toxic substances from the environment and serve as the structural basis for maintaining the permeability of the intestinal epithelium and its barrier effect (45, 48). The integral intestinal mechanical barrier functions by restricting the invasion of detrimental substances and microorganisms and primarily comprises enterocytes, goblet cells, M-cells, and intestinal chromaffin cells, which are sealed by tight junctions (Tjs), side channels that control the passage of molecules, ions, and water, effectively sealing out detrimental intestinal luminal substances from the intestines (4).

Intestinal goblet cells are directly involved in the composition, as well as the functioning of the intestinal mechanical barrier, by secreting intestinal *trefoil factor 3* (*TFF3*) and intestinal goblet cell specific protein *resistin-like molecule β* (*RELM β*), which are essential for maintaining the intestinal barrier integrity and stability and regulating colonic inflammatory susceptibility (4, 61–65). *TFF3*, a small peptide secreted by intestinal goblet cells, effectively improves the intestinal barrier function by regulating the TJs between intestinal epithelial cells to reduce the permeability of the paracellular pathway (4). In contrast, *RELM β* promotes increased *interleukin (IL) 22* by recruiting CD4 T lymphocytes, which subsequently promotes epithelial cell proliferation, indirectly mitigating intestinal damage in the disease and protecting the intestinal barrier function (61) (**Figure 3b**). Thus, intestinal goblet cells are crucial in the formation and functioning of the intestinal mechanical barrier.

### 4.3 Role of the intestinal goblet cells in the immune barrier

The intestine is an organ that contains the most number of immune cells, making it crucial for human immune function (66). The intestinal goblet cells participate in the function of the intestinal immune barrier (25). Studies have shown that intestinal goblet cells form goblet cell-associated antigen passages (GAPs), which transport luminal antigens to CD103^+^ dendritic cells and other mononuclear phagocytes in the lamina propria. Some of these cells migrate to draining lymph nodes to initiate adaptive immune responses and promote intestinal immune tolerance (26, 67).

In addition to the above-mentioned role of antigen delivery, intestinal goblet cells perform immune functions by synthesizing and secreting *RELMβ*. Artis et al. (68, 69) demonstrated that *RELMβ* exerts an inhibitory effect on gastrointestinal nematode infections by binding to the nematode chemosensory apparatus. Elevated *RELMβ* levels have been associated with a Th2-type polarized immune response, indicating that intestinal goblet cells may also participate in immunomodulation through *RELMβTh2 (*70). Similarly, Wang et al. (40) identified a potential new marker for goblet cells in the human intestines, *interectin-1* (*ITLN1*), when analysing the different nutrient absorption functions in the human intestines using a single-cell transcriptome. *ITLN1* was specifically expressed by all goblet cells in the ileum, colon, and rectum, whereas previous studies have shown that *ITLN1* binds to microbial glycans and functions in innate immunity, suggesting their role in innate immunity (71). Nyström et al. (36) found that intestinal goblet cells have a defence profile of expressing antimicrobial genes, implying that they may participate in the composition and functioning of the innate immune system. Moreover, the goblet cells regulate the immune response by secreting cytokines *IL6*, *IL7*, *IL13*, *IL15*, *IL17*, *IL18*, and *IL25*, as well as *chemokine exotoxin C-C motif chemokine ligand* (*CCL*) *6*, *CCL9*, and *CCL20 (*72) (**Figure 3c**). These findings suggest that in addition to secreting mucus, intestinal goblet cells function in regulating intestinal immune responses.

### 4.4 Role of intestinal goblet cells in the biological barrier

The colonic mucus layer contains two layers, primarily composed of highly glycosylated *MUC2*, in which the inner mucus layer impedes the penetration of bacteria and other harmful substances, separating the microbiota from the intestinal mucosa, preventing their direct contact that induces the occurrence of related diseases; whereas, the outer mucus layer is relatively sparse, allowing the entry of bacteria and other harmful substances, making it the primary habitat for commensal bacteria on the surface of the intestinal tract (3, 52, 73, 74). The biological barrier on the intestinal surface is a complex microbial system, and the outer mucus layer formed by intestinal goblet cells provides attachment sites and serves as a nutrient source, while these microorganisms may also influence goblet cell differentiation and mucin maturation, possibly affecting the maintenance of intestinal homeostasis (74, 75). Park et al. (76) found that *Bifidobacterium breve CBTBR* could effectively alleviate intestinal inflammation by promoting goblet cell regeneration, whereas *Bacillus subtilis* inhibited the Notch pathway and decreased *Hes1* expression in a Toll-like receptor 2-Myd88-dependent manner, causing the differentiation of intestinal stem cells (ISCs) towards the secretory spectrum (16). Troll et al. (77) demonstrated that microbiota-induced Myd88-dependent signalling pathways promote the differentiation of the intestinal secretory profiles by inhibiting the Notch signalling pathway, causing the intestinal goblet cells to interact with intestinal microbes to maintain intestinal homeostasis (**Figure 3d**).

## 5 Factors affecting intestinal goblet cells

### 5.1 Factors affecting intestinal goblet cell development

Intestinal goblet cells, as a type of secretory cells on the surface of the intestinal mucosa, are crucial in the formation and maintenance of the intestinal mucus barrier and the body’s immune response (4, 67). The developmental process of goblet cells involves multiple signalling pathways, which are simultaneously affected by various factors, including the Notch signalling pathway (11, 16, 76, 78–84), the Wnt/β-catenin pathway (30, 83, 85–93), the EGF pathway (30, 94), the Hippo pathway (31, 95–97), cytokines (11, 98–100), terminal differentiation-influencing factors such as *SPDEF* and *KLF4 (*32, 36, 72, 100–105), microorganisms, and energy metabolism (16, 36, 76–78, 106, 107). These signalling pathways and molecules play a role from the differentiation of ISCs into goblet cells to the stage of terminal differentiation and contribute to the overall development of goblet cells (**Table 2**).

**Table 2:** Factors affecting the development of goblet cells.

Classification			Mechanisms	Function	Ref.
Initial differentiation	Signalling pathway	Notch	–	Maintains intestinal goblet cell viability; critical in regulating intestinal goblet cell differentiation and development	(108)
		Wnt	–	Maintenance of intestinal secretion profile	(91)
			Promoting *Atoh1*^e^ expression	Prompting intestinal goblet cell differentiation	(93)
		EGF^a^	Interacting with *ADAM-17*^f^	Prompting intestinal goblet cell differentiation	(94)
			Interacting with the Wnt signaling pathway	Prompting intestinal goblet cell differentiation	(30)
		Hippo	–	Prompting intestinal goblet cell differentiation	(95)
	Cytokine	*IL* ^b^ *10*	Functioning through the *IL*^b^*10*-Notch axis	Inhibiting goblet cell differentiation	(11)
		*IL* ^b^ *33*	Promoting *IL*^b^*13* secretion	Prompting intestinal goblet cell differentiation	(98)
		*IL* ^b^ *17*	Promoting *Atoh1*^e^ expression	Prompting intestinal goblet cell differentiation	(99)
		*IL* ^b^ *18*	Regulating goblet cell transcription	Inhibiting goblet cell differentiation	(100)
Terminal differentiation		*AGR2* ^c^	–	Prompting goblet cell terminal differentiation	(101)
		*SPDEF* ^d^	Reducing the number of other epithelial cells	Prompting intestinal goblet cell terminal differentiation	(128)
		*OASIS*	–	Prompting intestinal goblet cell terminal differentiation	(102)

^a^ epidermal growth factor; ^b^ interleukin; ^c^ Anterior gradient 2; ^d^ SAM pointed domain ETS factor; ^e^ atonal bHLH transcription factor 1; ^f^ a disintegrin and metalloproteinase protein-17.

#### 5.1.1 Factors influencing initial differentiation

##### 5.1.1.1 Signalling pathways

The Notch signalling pathway is significant in the fate of intestinal epithelial cells by regulating their differentiation into absorptive or secretory lineage cells (90). The Notch signalling pathway maintains intestinal goblet cell viability and is critical in regulating intestinal goblet cell differentiation and development (108). VanDussen et al. (109) reported that inhibition of Notch signalling led to increased differentiation of intestinal goblet cells in mice, whereas overexpression of the intracellular domain of Notch in the intestinal epithelium resulted in a reduction of secretory cell populations (110)*. Hes1*, a major downstream transcriptional target of Notch, was shown to suppress goblet cell differentiation—*Hes1*-deficient mice exhibited upregulated expression of both goblet cell markers and *Atoh1 (*111). Similarly, *Atoh1*, another key downstream effector of Notch, has been established as essential for secretory lineage differentiation, with its overexpression promoting secretory cell fate (112, 113). However, apparent contradictions emerge regarding the regulatory relationships among these factors. While Notch signalling negatively regulates *Atoh1* and indirectly inhibits goblet cell differentiation via *Atoh1* suppression (95, 114–116), Kim et al. (117) proposed a more complex interaction: *Atoh1* may act upstream of Notch and directly repress *Hes1* expression, suggesting that *Hes1* functions downstream of *Atoh1* rather than solely as a direct Notch target. These seemingly conflicting results indicate that the regulatory network between Notch, *Atoh1*, and *Hes1* may be context-dependent, exhibiting feedback mechanisms or tissue-specific effects. Rather than a simple linear pathway, the differentiation of intestinal stem cells into goblet cells appears to be regulated through a dynamic interplay between Notch signalling and its downstream targets *Atoh1* and *Hes1*, with the latter also participating in cross-regulatory loops. Further studies are needed to clarify the precise hierarchical and temporal relationships under different physiological and experimental conditions. Furthermore, some molecules reportedly regulate goblet cell differentiation by altering the activity of the Notch pathway. By analysing the relationship between tryptophan metabolism and intestinal epithelial function, Alvarado et al. (79) showed that *indoleamine 2, 3-dioxygenase 1* inhibits the Notch signalling pathway by binding to the aryl hydrocarbon receptor, which promotes ISC differentiation into the secretory lineage cells. Moreover, other studies have demonstrated that *6formylindolo[3,2-b]carbazole*, the ultraviolet photoproduct of *L-tryptophan*, inhibits the Notch signalling pathway by activating *aryl hydrocarbon receptor-pErk1/2*, promoting goblet cell differentiation (118). These studies suggest that the Notch signalling pathway is crucial in the development of intestinal goblet cells.

In addition to the Notch signalling pathway, the Wnt/β-catenin pathway is significant in the determination of intestinal stem cell differentiation (90). Using transgenic mice ectopically expressing the Wnt secretory inhibitor *Dickkopf1*, Pinto et al. (91) found that Wnt deletion resulted in the loss of intestinal crypts, reduction of epithelial cell proliferation, and an essentially complete deletion of the intestinal secretory profile. Kuhnert et al. (92) demonstrated similar phenomena to those described above in intestinal epithelial cells by knocking down *Dickkopf1*. Furthermore, the Wnt/β-catenin pathway influences intestinal goblet cell development by interacting with effector molecules in the Notch signalling pathway (90). Kayet et al. (119) revealed that in the presence of Wnt, β-catenin inhibited Notch-mediated cellular oscillations induced by *Hes1* transcription by directly binding to the *Hes1* promoter and induced the cells to maintain a steady state. As a downstream of Notch, *Atoh1* is regulated by Notch and the Wnt/β-catenin signalling pathway. When β-catenin conduction is activated, *Atoh1* expression suppresses the forced differentiation induced by the Notch inhibition of *Atoh1* expression, and the upregulated *Atoh1* expression promotes goblet cell differentiation (93). Therefore, in addition to the Notch pathway, the development of intestinal goblet cells is simultaneously regulated by Wnt/β-catenin.

*EGF*, a cytoprotective peptide, participates in cell growth, proliferation, differentiation, and apoptosis, as well as in regulating the development of intestinal goblet cells (30, 94, 120–124). Shimoda et al. (94) demonstrated that the inhibition of *disintegrin and metalloproteinase-17* expression at the cellular level maintained and promoted goblet cell differentiation through a *disintegrin and metalloproteinase protein-17-EGFR* signalling. Furthermore, a recent study showed that *EGF* maintains and promotes goblet cell (e.g., piglets intestinal goblet cells) differentiation through the *EGFR* and Wnt/β-catenin signalling pathways (30).

As an evolutionarily highly conserved signalling pathway, the Hippo signalling pathway comprises an upstream signal, a central kinase core, and downstream target genes, and this pathway restricts tissue overgrowth, suppressing tumorigenesis primarily through the inactivation of its downstream effector protein *Yes-associated protein 1* (*YAP1*) and transcriptional coactivator with *PDZ-binding motif* (*TAZ*), and *YAP1/TAZ* participate in intestinal cell proliferation and differentiation (95, 125). Fallah et al. (95) found a marked increase in intestinal goblet and absorptive cell differentiation by knocking down *YAP1*, while *TAZ* knockdown revealed that *YAP1* exerted its inhibitory effect on intestinal goblet cell differentiation primarily through the intestinal transcription factor caudal-type homeobox transcription factor 2; thus, the Hippo pathway is involved in the regulation of intestinal stem cell activity and influences intestinal goblet cell differentiation (95, 126). Similarly, the Hippo pathway is associated with several other signalling pathways and affects *EGF* signalling pathway conductance by regulating *EGF* ligand expression and influences ISC activity by inhibiting the Wnt pathway activity. The Hippo pathway is also linked to the Notch signalling pathway (126, 127). Thus, the above pathways collectively regulate intestinal goblet cell development.

##### 5.1.1.2 Cytokines

In addition to relevant signalling pathways, specific cellular molecules participate in regulating the intestinal goblet cell differentiation process. Using zebrafish and mouse intestinal goblet organoid models, Rodrigo et al. (11) found that the inhibition of the *IL10*-Notch axis suppressed goblet cell differentiation. Through an intestinal immune cell co-culture model, Waddell et al. (98) demonstrated that *IL33* depended on the production of *IL13* by two groups of innate lymphoid-like cells, indirectly inducing intestinal goblet cell differentiation. Furthermore, using multiple conditional deletion models of *IL17*, Xun et al. (99) showed that *IL17* promoted differentiation into the intestinal secretory lineage, including intestinal goblet cells, by promoting *Atoh1* expression. However, Roni et al. (100) demonstrated that *IL18* inhibited intestinal goblet cell differentiation and maturation by regulating the transcriptional program of intestinal goblet cells. Therefore, the above studies confirm that many cytokines regulate intestinal goblet cell differentiation, improving the understanding of intestinal goblet cell development.

#### 5.1.2 Factors influencing terminal differentiation

Terminal differentiation of intestinal goblet cells is influenced by various factors, including the protein disulfide isomerase *Anterior gradient 2* (*AGR2*), *SPDEF*, the endoplasmic reticulum stress transducer OASIS, *bone morphogenetic protein* (*BMP*) signalling, the zinc-finger transcription factor *KLF4*, and *growth factor independence 1* (*Gfil1*) (36, 72, 101–104). Chen et al. (101) demonstrated that *AGR2* is essential for intestinal goblet cell terminal differentiation by knocking down and overexpressing *AGR2* in zebrafish. *SPDEF* acts as a downstream molecule of *Atoh1* and *Gfil1* to reduce Paneth and enteroendocrine cells to promote the terminal differentiation of goblet cells (128). By assessing the goblet cell markers in OASIS-knockdown mice, Asada et al. (102) found that the mature goblet cell marker *TFF3* exhibited a reduced expression, whereas the expression levels of the early goblet cell markers *MUC2*, *AGR2*, and *RELM β* were elevated, suggesting that OASIS regulates the developmental process of goblet cells primarily by affecting the terminal differentiation of goblet cells. Using *BMP*-knockout Bmpr1a mutant mice, Auclair et al. (103) revealed that *BMP* deficiency impairs the maturation of intestinal goblet cells by affecting their terminal differentiation. Analysis of *KLF4* showed that the number of mature goblet cells was reduced in *KLF4*-knockout mice, whereas that of goblet cells at the initial differentiation stage was not significantly affected, demonstrating that *KLF4* regulates terminal goblet cell differentiation (104). In addition, the terminal differentiation of intestinal goblet cells is reportedly regulated by specifically knocking down the molecular dedicator of the *cytokinesis 4* gene in mice by decreasing the degree of transcriptional maturation of goblet cells and inhibiting goblet cell maturation and *MUC2* production, and the dedicator of *cytokinesis 4* function is regulated through the *Gfil1*-*SPDEF* pathway (32). In addition to the molecules in the above studies, *IL18* affects the terminal differentiation of intestinal goblet cells. For example, Nowarski et al. (100) used *IL18*-deficient mice to show that *IL18* inhibits the terminal differentiation of goblet cells and affects their maturation, contributing to the development of associated intestinal diseases. Hood et al. (105) found that the terminal differentiation of intestinal goblet cells was affected by knocking down *tumor necrosis factor-alpha-induced protein 8* in mice and also identified a novel *Nupr1*, a new regulatory factor that may affect the terminal differentiation of intestinal goblet cells; however, the specific mechanism by which they affect the terminal differentiation of goblet cells requires further investigation. The above studies suggest that the terminal differentiation of intestinal goblet cells is influenced by several factors that regulate their functional performance by promoting or inhibiting their maturation.

#### 5.1.3 Other factors

In addition to the above pathways and associated molecules that regulate the intestinal goblet cell differentiation process, other factors, such as microorganisms, influence goblet cell differentiation. For example, Troll et al. (77) found that the microbiota-induced signalling pathway promotes the differentiation of gut secretory lineage by inhibiting the Notch signalling pathway. In addition, energy metabolism affects goblet cell differentiation, and Marlies et al. (78) recently demonstrated that Forkhead box O increases mitochondrial fission by interacting with Notch, a phenomenon mediated by *miR484* and *FIS1*, specifically leading to increased levels of secretory cells without affecting those of absorptive cells. In addition, Shijie et al. (106) showed that tissue sclerosis affects intestinal goblet cell differentiation when they observed the characteristics of patients with IBD, suggesting that it promotes the expansion of *Olfactomedin4+* cells to villus-like regions by increasing *YAP* expression while inducing the nuclear translocation of *YAP*, benefiting the preferential differentiation of ISCs to goblet cells. Similarly, single-cell RNA sequencing and proteomic analysis of intestinal samples from mice and humans revealed the specific markers and transcription factors enriched in intestinal goblet cells, including *breast carcinoma amplified sequence 1*, *serine protease inhibitor Kazal-type 4*, *REP15*, *cAMP-responsive element binding protein 3 L1*, and forkhead box A3; however, the effects of these factors on intestinal goblet cell development require further investigation (36, 107).

### 5.2 Factors affecting the function of intestinal goblet cells

The intestinal mucus barrier is considered the first defence line against harmful substances, and as intestinal goblet cells are crucial in it, understanding the various factors that influence their function is essential. Naama et al. (129) analysed autophagy-initiating protein *Beclin 1*-knockout mice and found that *Beclin* activated the onset of autophagy, causing a reduction in endoplasmic reticulum stress and promoting the intestinal goblet cells to produce more mucus to maintain intestinal mucus barrier integrity under inflammatory conditions. Engevik et al. (47) demonstrated that the products secreted by *Bifidobacterium*, particularly *γ-glutamylcysteine*, promote the mucus secretion of intestinal goblet cells by inhibiting the *ROS* and *NF-κB* driving the endoplasmic reticulum stress while reducing the number of pro-inflammatory factors. Studies have shown that the transcription factor *NF-κB* increases the number of goblet cells; thus, *γ-glutamylcysteine* and *NF-κB* jointly promote the maintenance of intestinal homeostasis by affecting the intestinal goblet cell function (130). Furthermore, Pavel et al. (131) showed that long noncoding RNA-H19 impairs intestinal barrier function by inhibiting intestinal goblet and Paneth cell functions, as well as inhibiting the occurrence of intestinal autophagy.

*MUC2*, a major mucin secreted by intestinal goblet cells, is also crucial for intestinal goblet cell function. Shuailing et al. (132) found that somatostatin in mice promotes *MUC2* secretion from intestinal goblet cells through the Notch-*Hes1* pathway. Michael et al. (133) showed that *ERN2/IRE1β*, an analogue of the endoplasmic reticulum pressure sensor *ERN1/IRE1α*, forms an intestinal chemical barrier by modulating the maturation of intestinal goblet cells and their secretion of mucins.

In addition to these influences, signalling pathways regulate intestinal goblet cell function. For example, Nicolás et al. (134) found that defects in *BMPs* cause impaired intestinal goblet cell function, affecting the integrity of the mucus barrier. Min et al. (135) showed that histamine derived from conjunctival goblet cells promotes mucin production through the activation of *EGFR*. Mira et al. (136) demonstrated that *EGF* improved intestinal goblet cell-associated mucosal integrity. In addition, Wu et al. (137) found that *Clostridium butyricum*, a probiotic that produces butyrate, induces *EGFR* activation via its ligands, causing elevated levels of intestinal goblet cells and *MUC2* secretion, corroborating the findings of Min et al. and Mira et al. above. Thus, intestinal goblet cells are regulated by various factors in the formation and maintenance of the intestinal mucus barrier, influencing intestinal homeostasis and mediating the development of different intestinal diseases.

In addition to their ability to form and maintain the intestinal chemical barrier, intestinal goblet cells function in immunomodulation. Through a specific *lymphotoxin β receptor* (*LTβR*) knockdown in mice, Yaya et al. (138) showed that *LTβR* is crucial for intestinal goblet cell proliferation and *MUC2* expression during intestinal infection with Listeria monocytogenes and further analysed that single gene-deficient mice were informed that Type 3 Innate Lymphoid Cells through the lymphotoxin*LTβR* pathway to promote intestinal goblet cells differentiation and function as well as *MUC2* expression, facilitating the host to improve intestinal defence function. The maintenance of homeostasis between gut microbes and the host is pivotal for the protection of intestinal health (139). Gao et al. (140) found that dysregulated microbiota, particularly *E. coli*, influences *gasdermin D* activation during colitis, while Roni et al. (100) showed that *gasdermin D* reduces the number of intestinal goblet cells by promoting *IL18* secretion, which contributes to intestinal disease. In addition, a recent study revealed that *IL18* of enteric nervous system origin influences the function of the intestinal immune system by promoting the secretion of antimicrobial peptides by intestinal goblet cells and that it modulates the intestinal mucosal barrier defence against bacterial invasion (141). Therefore, intestinal goblet cell function is influenced by various factors, and these data can provide insights into identifying novel approaches for treating intestinal disorders that are associated with abnormal intestinal goblet cell function.

## 6 Intestinal goblet cells and IBD

**Figure 4:**
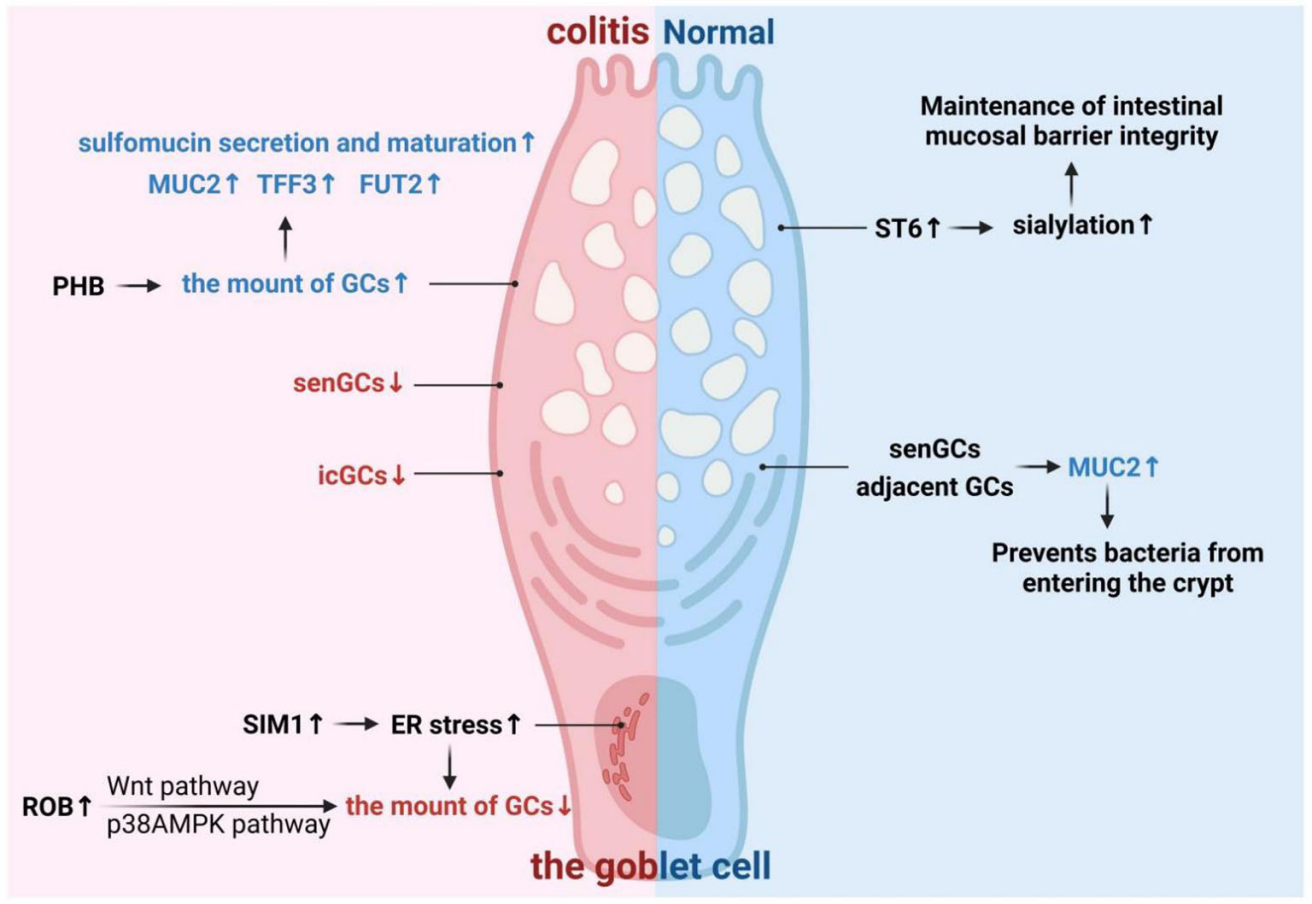
Relationship between intestinal goblet cells and inflammatory bowel disease (IBD). As a member of the intestinal epithelial cells, the changes in the function or number of intestinal goblet cells cause the occurrence of intestinal diseases including IBD. senGC, Sentinel goblet cell; MUC2, Mucin 2; ST6, ST6GALNAC1; TFF3, Trefoil factor 3; FUT2, Fucosyltransferase 2; PHB, Poly-β-hydroxybutyrate; icGCs, Intercrypt goblet cells; SIM1, Stromal interaction molecule 1; ER, Endoplasmic reticulum.

Inflammatory bowel disease (IBD), which mainly includes ulcerative colitis (UC) and Crohn’s disease (CD), is a chronic inflammatory condition of the digestive tract marked by recurring flares and a complex, still not fully understood cause (142). While immune dysfunction has long been considered a main driver, more and more evidence points to primary defects in the intestinal epithelial barrier-especially problems with goblet cells-as major contributors to the disease (5, 8).As the primary mucus producers in the gut lining, goblet cells are key to building the protective layer that keeps microbes away from the epithelial surface. Goblet cell dysfunction or depletion is a known characteristic of IBD (**Figure 4**), but the specifics and severity of these issues vary significantly between UC and CD (73, 143, 144).

### 6.1 Distinct patterns of goblet cell dysfunction in ulcerative colitis and Crohn’s disease

UC and CD both have goblet cell abnormalities, but the root causes and how they show up are not the same (1, 5, 8, 145, 146). **Table 3** breaks down the main differences in goblet cell pathology between the two. Gersemann et al. (145) dug into these differences by looking at colon samples from UC and CD patients with similar levels of inflammation. They found that inflammation ramps up the expression of goblet cell differentiation factors *Atoh1* and *KLF4* in CD, but not in UC (145). They also checked protein levels and got the same result for *Atoh1* (145). Even with similar inflammation levels, both diseases lost goblet cells, but in UC, the loss was mainly in the top part of the crypts. This points to a problem with cells finishing their development, not just failing to become goblet cells in the first place (5, 145). For ileal CD, the main issue is with Paneth cells, not goblet cells. Their defensin levels drop because Wnt signalling is off, which throws off Paneth cell development (5, 8). Without enough defensin, the antimicrobial barrier weakens and bacteria can get through. So, UC has a mucus problem from faulty goblet cell development, while ileal CD has a chemical barrier problem from Paneth cells not doing their job (5, 8).

**Table 3:** Unique characteristics of goblet cell malfunction in ulcerative colitis and Crohn’s disease.

Feature	Ulcerative Colitis (UC)	Crohn’s Disease (CD)	Ref.
Goblet cell number	Markedly reduced, particularly in active disease	Generally preserved; may be decreased in inflamed areas but less severe than UC	(1, 5, 145)
Mucus layer thickness	Thinner, discontinuous, and denuded in parts	Relatively preserved, though compositional changes may occur	(5, 8, 146)
Differentiation defect	Impaired terminal differentiation; failure to induce *Atoh1*^a^ and *KLF4*^b^ in response to inflammation	Enhanced goblet cell differentiation in response to inflammation (*Atoh1*^a^ and *KLF4*^b^ induced)	(5, 145)
Histological distribution	Goblet cell depletion most pronounced in the upper third of colonic crypts	Goblet cells present throughout crypts, though crypt architecture may be distorted	(1, 145)
MUC2 expression	Decreased *MUC2*^c^ production; altered glycosylation patterns	*MUC2*^c^ expression relatively maintained, but defensin deficiency in Paneth cells (ileal CD)	(8, 145, 146)
Associated epithelial defect	Primary goblet cell differentiation failure	Paneth cell dysfunction (ileal CD); defensin deficiency	(5, 8)
Inflammatory response to barrier defect	*Th*^d^*2*/*Th*^d^*9*-mediated inflammation	*Th*^d^*1*/*Th*^d^*17*-mediated inflammation with defective innate immunity	(5, 8)

^a^ atonal bHLH transcription factor 1; ^b^ Krüppel-like factor 4; ^c^ Mucin 2; ^d^ interleukin.

### 6.2 Microbiota alterations and their impact on goblet cells and the mucus barrier

The gut microbiota has a dual function in the development of IBD: while commensal bacteria are crucial for preserving epithelial balance, an imbalance in microbial composition, known as dysbiosis, can compromise the mucus barrier and worsen goblet cell dysfunction (74, 75, 139).

#### 6.2.1 Mucus layer as a microbial habitat

The outer mucus layer acts as a main habitat for commensal bacteria, offering places to attach and a source of nutrients (74). These microorganisms, in turn, affect the differentiation of goblet cells and the maturation of mucin. Research has indicated that specific types of bacteria can alter goblet cell function via various methods: bifidobacterium breve aids in the regeneration of goblet cells and reduces intestinal inflammation; bacillus subtilis suppresses the Notch signalling pathway and reduces *Hes1* expression through a TLR2-MyD88-dependent mechanism, promoting ISC differentiation into the secretory lineage; clostridium butyricum triggers EGFR activation through its ligands, leading to an increase in goblet cell numbers and *MUC2* secretion (16, 76, 137). On the flip side, bad bacteria mess with the mucus barrier. When colitis flares up, E. coli moves in and messes with gasdermin D. That cranks up IL-18 and wipes out goblet cells (100, 140).

#### 6.2.2 Dysbiosis in CD and UC

The gut microbiota composition varies between CD and UC patients, affecting the mucus barrier differently (139) (**Table 4**).

**Table 4:** Dysbiosis in ulcerative colitis and Crohn’s disease.

Feature	Ulcerative colitis (UC)	Crohn’s disease (CD)
Microbial signature	Reduced microbial diversity; decreased Firmicutes; increased Proteobacteria	More pronounced dysbiosis; reduced Faecalibacterium prausnitzii (antiinflammatory species)
Mucus interaction	Bacteria penetrate the inner mucus layer due to barrier thinning	Adherent-invasive E. coli (AIEC) adhere to and invade epithelial cells
Effect on goblet cells	Microbial products induce ER stress and apoptosis	Bacterial adherence triggers inflammatory responses that indirectly affect goblet cells

#### 6.2.3 Mucus glycosylation and bacterial interactions

The effectiveness of mucus protection is heavily reliant on its glycosylation status. Yao et al. (146) showed that sialylation is vital in maintaining the integrity of the intestinal mucus barrier and preventing bacterial invasion. Whereas *ST6GALNAC1* (*ST6*), the rate-limiting step in salivary acylation, is highly expressed in intestinal goblet cells, and *ST6* deficiency causes decreased sialylation, leading to impaired intestinal mucus barrier integrity and early onset of IBD (146). By screening a national IBD cohort, Yao et al. (146) found that *ST6* mutations are associated with the development of “very early-onset IBD,” and therefore, intestinal goblet cells participate in the development of IBD through the high expression of ST6. In addition, Yao et al. (146) speculated that the pathogenesis of IBD may be associated with changes in the biochemical composition of the intestinal mucus barrier, particularly sialylation and epithelial cell changes, as suggested by the present study and many previous reports (146). Therefore, it is proposed that in addition to anti-inflammatory and immunotherapeutic treatments, IBD treatment should consider therapeutic tools related to intestinal barrier and epithelial cell repair, providing a novel approach for IBD treatment (146). In a similar way, the maturation of sulfomucin by goblet cells aids in the function of the mucus barrier. Poly-β-hydroxybutyrate (PHB) eases colitis by boosting the secretion and maturation of sulfomucin, indicating that modifying mucus biochemistry could be a therapeutic strategy (147).

### 6.3 Integrated view: goblet cells, mucus barrier, tight junctions, and microbiota in IBD pathogenesis

The intestinal barrier consists of multiple layers, including: (1) the mucus layer, which acts as a chemical barrier, (2) the epithelial cell layer with tight junctions, serving as a mechanical barrier, (3) immune cells and their secretions (immune barrier), and (4) the resident microbiota (biological barrier) (45, 48). Goblet cells play a role in all four layers by secreting mucus, producing *TFF3*, sampling antigens (GAPs), and secreting cytokines. Nevertheless, their role cannot be viewed independently (4, 26, 67).

#### 6.3.1 Tight junctions and paracellular permeability

Intestinal epithelial cells are connected by tight junctions, which create selectively permeable barriers to control paracellular transport. Recent progress has revealed three unique permeability routes through TJs: the pore pathway, which is selective for size and charge; the leak pathway, which permits larger molecules; and the unrestricted pathway, found at sites of epithelial damage (2, 4). In IBD, all three pathways have shown increased permeability (2). Significantly, primary TJ dysfunction can trigger mucosal immune responses and hasten the development of immune-mediated colitis, even without obvious inflammation (4).

*TFF3* from goblet cells is essential for maintaining the integrity of tight junctions (4). It improves barrier function by influencing the expression and placement of tight junction proteins, thereby decreasing paracellular permeability (4). Therefore, goblet cell dysfunction in IBD not only weakens the mucus barrier but also worsens TJ defects, leading to a combined barrier failure.

#### 6.3.2 A unifying view of barrier failure in IBD

Initial defects in certain epithelial lineages trigger unique pathological sequences in inflammatory bowel disease. In ulcerative colitis, the final differentiation of goblet cells is disrupted, resulting in decreased *MUC2* production and changes in mucus characteristics (3, 5). In ileal Crohn’s disease, the malfunction of Paneth cells leads to a lack of defensins, weakening the antimicrobial barrier (5, 8). The mucus barrier is compromised by these epithelial abnormalities, which permits luminal bacteria to infiltrate the inner mucus layer and make direct contact with the intestinal lining (73, 146).

Dysbiosis worsens this issue. Harmful bacteria like adherent-invasive Escherichia coli in Crohn’s disease attach to and invade epithelial cells, whereas beneficial bacteria that typically aid goblet cell function and mucus production are depleted (16, 76, 137). The resulting epithelial stress responses, such as endoplasmic reticulum stress and oxidative stress, further damage goblet cell function and trigger apoptosis, worsening the initial issue (47, 129, 148).

At the same time, the disruption of tight junctions enhances paracellular permeability, permitting bacterial products and luminal antigens to reach the lamina propria (2, 4). *TFF3* from goblet cells, which typically maintains tight junction integrity, is reduced when goblet cells malfunction, leading to a combined breakdown of the mucus barrier and paracellular seal (4). The breach of the epithelial barrier that results lead to immune activation, causing inflammatory damage that continues the cycle and further impairs epithelial function (5, 8).

In IBD pathogenesis, goblet cell dysfunction, mucus barrier defects, tight junction abnormalities, and dysbiosis are interlinked and reinforce each other. If the fundamental barrier defects remain, therapeutic approaches focusing solely on one aspect, like immunosuppression, might be inadequate. The inadequate reaction of VEO-IBD patients lacking IL10RA to standard anti-inflammatory treatments highlights the critical necessity for methods that repair epithelial barrier integrity (6, 11).

### 6.4 Therapeutic implications

Recognition of the multifaceted role of goblet cells and the mucus barrier in IBD opens new therapeutic avenues (**Table 5**).

**Table 5:** Therapeutic implications.

Target	Approach	Examples	Ref.
Goblet cell differentiation	Enhance *Atoh1*^a^/*KLF4*^b^ expression; modulate Notch/Wnt/Hippo pathways	*SPINK4*^c^ (activates EGFR^d^-Wnt-Hippo); *YAP1*^e^ inhibition	(95, 149–151)
*MUC2*^f^ production/secretion	Promote mucin synthesis and exocytosis	PHB^g^ (enhances sulfomucin); β-hydroxybenzoic acid	(60, 147)
Mucus glycosylation	Restore normal sialylation patterns	*ST6*^h^-targeted therapies	(146)
Tight junction integrity	Stabilize TJs^i^; regulate *MLCK*^j^ activity	*TFF3*^k^ supplementation; *MLCK*^j^ inhibitors	(4, 152)
Microbiota modulation	Restore beneficial bacteria; correct dysbiosis	Bifidobacterium; Clostridium butyricum; fecal microbiota transplantation (ileal CD)	(76, 137)
ER^l^ stress reduction	Alleviate goblet cell ER^l^ stress	γ-glutamylcysteine; *STIM1*^m^ inhibition	(47, 148)

^a^ atonal bHLH transcription factor 1; ^b^ Krüppel-like factor 4; ^c^ serine protease inhibitor Kazal-type 4; ^d^ epidermal growth factor receptor; ^e^ Yes-associated protein 1; ^f^ Mucin 2; ^g^ Poly-β-hydroxybutyrate; ^h^ ST6GALNAC1; ^i^ Tight junction; ^j^ Myosin Light Chain Kinase; ^k^ Trefoil Factor 3 ^l^ Endoplasmic Reticulum; ^m^ Stromal interaction molecule 1.

### 6.5 Conclusions and future directions

Intestinal goblet cells are key to keeping the multilayered gut barrier in good shape. When they don’t work right, it feeds into IBD in a few ways-like not making enough mucus, messing up mucin sugar patterns, messing with antigen sampling, and throwing off talks with the microbiome. Worth noting, how goblet cells go wrong isn’t the same in UC and CD. In UC, the main hiccup is at the final stage of cell maturation, while in CD, the epithelial issues run wider, even pulling in Paneth cells. These differences actually matter when it comes to treatment angles. Getting a solid grip on goblet cell biology-especially how it ties into the mucus layer, tight junctions, and gut bugs-opens up fresh ways to fix the intestinal barrier in IBD. It’s about moving past just calming the immune system and aiming for real repair and lasting balance.

## 7 Conclusions

Intestinal goblet cells, a specialized epithelial cell type, play essential role in forming the protective mucus barrier via *MUC2* secretion, which blocks pathogenic invasion and supports commensal microbes. Recent research reveals that goblet cells contribute not only to the mechanical barrier but also to immune and biological barrier functions, highlighting their broader role in mucosal immunity. Their remarkable plasticity and heterogeneity enable adaptive responses to environmental cues, thereby preserving intestinal integrity under physiological and pathological conditions. This review summarized advances in goblet cell differentiation, classification, functions, and their involvement in IBD, offering new insights into their role in intestinal health.

Emerging evidence indicates that dysfunction or depletion of intestinal goblet cells serves as a critical hallmark of IBD, leading to barrier impairment, microbial dysbiosis, and sustained inflammation. The identification of distinct goblet cell subtypes offers novel potential targets for IBD intervention. Meanwhile, signalling pathways and various molecules involved in regulating goblet cell differentiation also provide new insights for the treatment of IBD. Future translational research should focus on developing small molecules or biologics that can modulate specific goblet cell subtypes or their key regulatory pathways (e.g., boosting *MUC2* production via *Atoh1*, or targeting senGC function). Additionally, goblet cell-derived molecules in stool or blood could be explored as biomarkers for early diagnosis or monitoring treatment response in IBD.

Therefore, a profound understanding of goblet cell biology may pave the way for novel therapeutic strategies aimed at restoring intestinal function in IBD and other gut disorders by targeting their multifaceted roles—such as mucus production, antigen sampling, and cytokine secretion. Developing targeted therapies based on these mechanisms holds significant potential for bridging basic research and clinical applications.

## Glossary

IBD: inflammatory bowel disease
*MUC2*: *mucin 2*
*Atoh1*: *atonal bHLH transcription factor 1*
EGF: epidermal growth factor
*Hes1*: *hairy and enhancer of split-1*
*AGR2*: *Anterior gradient 2*
BMP: bone morphogenetic protein
LTβR: lymphotoxin β
PHB: poly-β-hydroxybutyrate
*KLF 4*: *Krüppel-like factor 4*
*SPDEF*: *SAM pointed domain ETS factor*
senGCs: sentinel goblet cells
clca1: calcium-activated chloride channel regulator 1
Fcgpb: IgGFc-binding protein
*Gsdmc 4*: *Gasdermin 4*
*Dmtb1*: *deleted in malignant brain tumors 1*
icGCs: intercrypt goblet cells
*ITLN*: *interectin-1*
*CCL*: *C-C motif chemokine ligand*
ISCs: intestinal stem cells
*YAP1*: *Yes-associated protein 1*
*TAZ*: *transcriptional coactivator with PDZ-binding motif*
*Gfil1*: *growth factor independence 1*
*ST6*: *ST6GALNAC1;TFF3*, *Trefoil Factor 3*.
